# A Review of Recent Advances in Deep Learning Models for Chest Disease Detection Using Radiography

**DOI:** 10.3390/diagnostics13010159

**Published:** 2023-01-03

**Authors:** Adnane Ait Nasser, Moulay A. Akhloufi

**Affiliations:** Perception, Robotics and Intelligent Machines Research Group (PRIME), Department of Computer Science, Université de Moncton, Moncton, NB E1C 3E9, Canada

**Keywords:** radiography, chest X-ray, computer-aided detection, machine learning, deep learning, deep convolutional neural networks

## Abstract

Chest X-ray radiography (CXR) is among the most frequently used medical imaging modalities. It has a preeminent value in the detection of multiple life-threatening diseases. Radiologists can visually inspect CXR images for the presence of diseases. Most thoracic diseases have very similar patterns, which makes diagnosis prone to human error and leads to misdiagnosis. Computer-aided detection (CAD) of lung diseases in CXR images is among the popular topics in medical imaging research. Machine learning (ML) and deep learning (DL) provided techniques to make this task more efficient and faster. Numerous experiments in the diagnosis of various diseases proved the potential of these techniques. In comparison to previous reviews our study describes in detail several publicly available CXR datasets for different diseases. It presents an overview of recent deep learning models using CXR images to detect chest diseases such as VGG, ResNet, DenseNet, Inception, EfficientNet, RetinaNet, and ensemble learning methods that combine multiple models. It summarizes the techniques used for CXR image preprocessing (enhancement, segmentation, bone suppression, and data-augmentation) to improve image quality and address data imbalance issues, as well as the use of DL models to speed-up the diagnosis process. This review also discusses the challenges present in the published literature and highlights the importance of interpretability and explainability to better understand the DL models’ detections. In addition, it outlines a direction for researchers to help develop more effective models for early and automatic detection of chest diseases.

## 1. Introduction

CXR imaging is a fast and cost-effective technique widely used by radiologists to diagnose multiple parts of the human body such as heart, lungs, bones, blood vessels, and airways [1]. It plays a major rule in detecting diseases and abnormalities. CXR images are typically generated by projecting X-ray radiation through the body positioned against the metallic plate of the X-ray machine. The organs appear differently on the CXR image because of the amount of radiation absorbed by each organ. The organs that absorb more radiation (e.g., bones) appear in white color, while the parts that absorb less radiation (e.g., heart) appear in different shades of gray. The airways and the organs containing air (e.g., lungs) appear in a black color [2]. CXR examinations are affordable, non-invasive and painless. They are considered as a valuable tool for the detection of many diseases and abnormalities, which helps in diagnosing diseases and monitoring therapy [3].

Chest diseases are the most common and dangerous health issues worldwide. Many people die from chest diseases every day, especially from lung cancer, pneumonia, tuberculosis (TB), and COVID-19 [4,5,6]. Chest diseases are fatal if not detected at their earlier stages. According to the WHO (World Health Organization), chest diseases have a very high mortality rate, and they can lead to death in several situations. As reported by WHO, an estimated 65 million people worldwide have COPD (chronic obstructive pulmonary disease), including 3 million deaths [7]. For pneumonia, the mortality rate is worrying, as it killed 808,694 children under the age of 5 in 2017 [8,9]. Around 10 million people fell ill with TB (1.2 million children, 3.2 million women, and 5.6 million men) with 1.4 million deaths [10]. The same is true for lung cancer, which kills approximately 1.6 million people annually [11].

In order to diagnose the patients, radiologists inspect visually the CXR images. This process is time and resource intensive, especially in areas where there is a shortage of qualified clinicians. The lower resolution of CXR images, the similarities between the signs of diseases, and the lack of experience and focus while examining a CXR image can make the interpretation a challenging task for radiologists as it can lead to potentially life-threatening diagnostic errors. Therefore, computer-aided detection systems (CAD), including computer vision, machine learning (ML) and deep learning (DL) algorithms, were proposed to provide a good decision-making tool for radiologists to diagnose different diseases [12,13,14].

For nearly a decade, ML techniques became more popular for medical imaging-based anomaly detection and classification, especially with the release of several datasets. These techniques were applied for various purposes in medical image analysis such as organs segmentation, diseases detection and classification. They showed high performance through numerous studies developed to classify several diseases such as TB, pneumonia, edema, cardiomegaly and COVID-19. For instance, Rasheed et al. [15] examined the use of ML for the diagnosis of COVID-19 using a logistic regression classifier with CXR images. They considered a dimensionality reduction approach to speed up learning and to obtain the highest possible accuracy (ACC) by selecting the most relevant features.

Elaziz et al. [16] proposed an ML method to classify CXR images into normal and COVID-19. FrMEMs (fractional multichannel exponent moments) technique was employed for features extraction, an MRFO (modified version of manta ray foraging optimization) method was used for features selection and KNN (k-nearest neighbors) classifier was employed to classify CXR images. Candemir et al. [17] used an algorithm that has two main stages for the cardiomegaly classification. The first is heart and lung region localization on CXR images, where the second is based on radiographic index extraction from lungs and heart edges. Alslatie et al. [18] proposed an SVM (support vector machines) algorithm for the classification of atelectasis and cardiomegaly. CXR images were segmented to localize the region of interest (ROI) and then enhanced by using gray-level transformation techniques. Avni et al. [12] worked on the classification of the cardiomegaly, pleural effusion, and septum enlargement. They used a non-linear multiple SVM algorithm to identify the manifested diseases. Sara et al. [19] used an SVM classifier to detect pneumonia using a pediatric dataset. Chandra and Verma [20] proposed an ML paradigm to detect pneumonia on segmented images. Five classifiers were used named random forest, logistic regression, SMO (sequential minimal optimization), multi-layer perceptron and classification via regression. Sousa et al. [21] used five ML classifiers (multi-layer perceptron, decision tree, naive Bayes, SVM, and KNN) combined with three techniques of dimensionality reduction (principal component analysis, sequential forward selection, and kernel principal component analysis) to detect childhood pneumonia. Varela-santos and Melin [22], proposed an ML features-based approach for the classification of pneumonia. Texture features were obtained using GLCM (gray level co-occurrence matrix) algorithm, and then the classification of images into normal or pneumonia was performed. Pavithra and Pattar [23] introduced an algorithm to detect and classify CXR images into pneumonia or lung cancer. The algorithm employs power law transform and median filter for noise removing, extracts the features using a Gabor filter, then performs a classification using a feed forward and radial basis function. A distance of two probability distributions algorithm namely, EMD (earth movers distance) was used by Khatri et al. [24] to extract the difference between two CXR images and detect whether it is a pneumonia or a non-pneumonia image. Subhalaxmi et al. [25] used three different machine learning techniques (LR (Logistic Regression), NN (Neural Network), and SVM) to predict pneumonia on CXR images. An extraction of features was performed using a GLCM algorithm. Inbaraj et al. [26] used a decision tree model called stacked loopy decision tree (SLDT) classifier with an ROI based approach to detect TB. Their proposed approach is based on three steps to distinguish TB, including segmentation, feature extraction, and classification. For more ML works, readers can consult the survey by Rahamat et al. [27] which reviews the use of ML methods for CXR classification and detection and summarizes the obtained results.

While ML models require users and data scientists to select features from the input data, DL models perform automatic features extraction. ML algorithms reveal less performance using large datasets, while DL algorithms perform better with the availability of large quantities of data and higher computational power [28]. Therefore, researchers are focusing more on DL techniques to increase the performance of medical applications and decrease the time and cost of the diagnostic process.

Multiple reviews were published presenting the application of DL techniques in medical image analysis for the detection of different diseases. Alghamdi et al. [29] reviewed convolutional neural networks and other deep learning techniques employed for the detection of COVID-19 using CXR images. Various CXR COVID-19 datasets were introduced and discussed in addition to numerous architectures proposed to automate the detection of COVID-19. They covered and highlighted different challenges facing the discussed DL approaches and datasets. Chandrasekar [30] explored the application of DL techniques for the detection of coronavirus in CXR images. Many papers presenting new DL approaches for features extraction and detection of coronavirus were outlined. They introduced the used CXR coronavirus datasets and analyzed the performance of DL models. Shyni and Chitra [31] presented a comparative study of preprocessing and deep learning techniques used for the automatic detection of COVID-19 on X-ray and computed tomography (CT) images. They highlighted the importance of transfer learning and data-augmentation techniques for the scarcity of COVID-19 datasets. Jiechao et al. [32] focused on DL techniques applied to detect four pulmonary diseases (pulmonary nodule, pulmonary embolism, pneumonia, and interstitial lung disease). They presented several DL frameworks used for medical images and discussed their architectures.

Most of these studies focus only on the application of DL models for COVID-19 detection and do not consider other diseases. In addition, many open access datasets are missing, as well as recent CXR image processing techniques that have shown a favorable effect on the performance of recent DL models. As far as we know, our review is the first to present all accessible collections of CXR images, including COVID-19 datasets. In comparison to prior reviews, our paper contributes as follows:

- It describes a total of 22 publicly available datasets containing CXR images from different institutions.
- It introduces commonly used processing techniques, and recently published research related to the automatic detection of various chest diseases (pneumonia, pulmonary nodules, tuberculosis, COVID-19, etc.) using radiological medical images and DL techniques.
- It highlights the necessity of using preprocessing and data-augmentation techniques to improve the quality of CXR images, solve data balance problems, and therefore increase the performance of the models used for chest disease detection.
- It discusses various concerns facing the research community, highlights the limitations of published studies, and suggests alternatives to help overcome these challenges.
- It presents recent published papers (the majority of them are between 2019 and 2022) and allows researchers to have easy access to state-of-the-art works.

**Figure 1:**
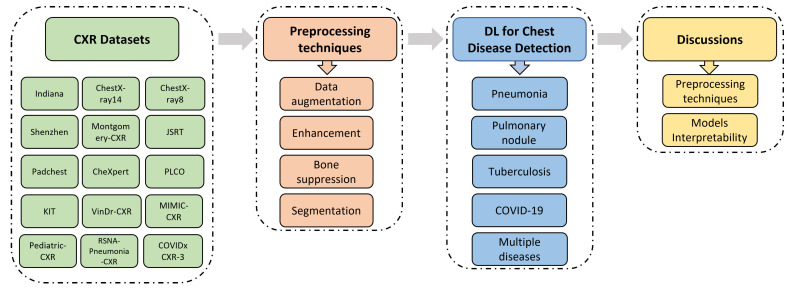
Structure diagram illustrating the topics discussed in our paper.

As depicted in Figure 1, this paper is structured as follows: Section 2 introduces the most widely used and publicly available CXR datasets. Section 3 illustrates the evaluation metrics and the most efficient preprocessing techniques for CXR medical images, including data-augmentation, image enhancement, bone suppression, and organs segmentation. Section 4 demonstrates recently published studies that have used DL techniques to detect and classify chest diseases. The results obtained by each of the mentioned papers are presented in tables grouped by diseases. Section 5 covers main challenges that most of the published papers have faced and discusses alternative solutions to be considered in future works in order to achieve more complete and relevant results. Finally, Section 6 concludes this work.

## 2. Datasets

**Figure 2:**
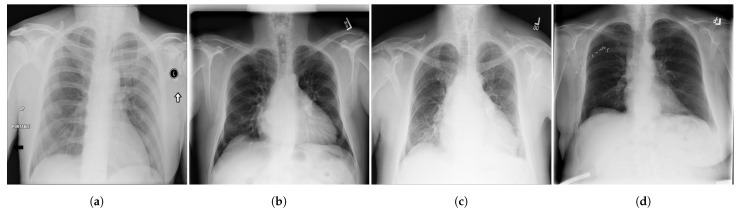
Examples of CXR images from ChestX-ray14 dataset [39] where, (**a**) Nodule; (**b**) Emphysema; (**c**) Effusion; (**d**) Infiltration.

**Figure 3:**
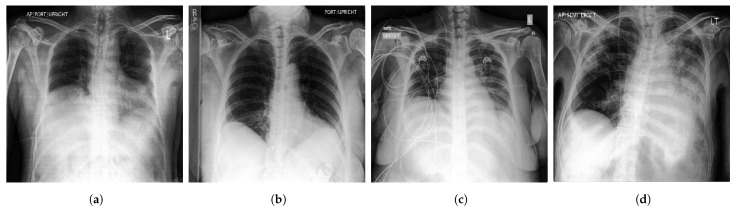
Samples of CXR images from CheXpert dataset [44] where, (**a**) Atelectasis; (**b**) Cardiomegaly; (**c**) Edema; (**d**) Pneumonia.

**Figure 4:**
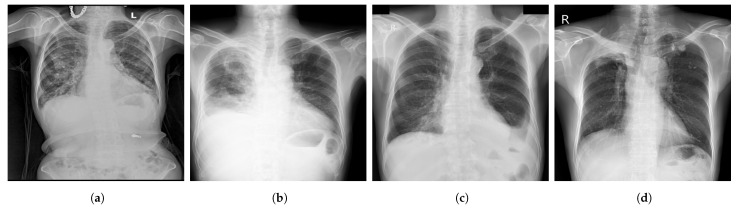
Examples of CXR images from VinDr-CXR dataset [48] where, (**a**) Infiltration; (**b**) Pleural Effusion; (**c**) Pneumothorax; (**d**) Pulmonary Fibrosis.

In the medical area, there are several types of image screening technologies, including ultrasound imaging, CT (computed tomography), MRI (magnetic resonance imaging), and X-ray imaging. Radiologists use these images to diagnose organs for the detection of abnormalities [33]. Detecting diseases from CXR images is always a difficult task for radiologists and sometimes leads to misdiagnoses. To address this purpose using CAD systems, a large amount of data is required for training and testing. CAD systems in medical analysis are usually trained and tested on an ensemble of data called a dataset, that are generally composed of images and other important information called metadata (e.g., age of patient, race, sex, Insurance type). Some hospitals, universities and laboratories in different countries used several approaches to collect data that belong to patients [34]. Datasets collection in medical area aims to advance research in detecting diseases. DL techniques proved their efficiency and ability to detect most dangerous diseases using different datasets [35,36]. These techniques achieved expert-level performance on clinical tasks in many studies [6,37]. There are multiple datasets that contain thousands of CXR images. Details about datasets are presented in Table 1 and Table 2. In this review, we are focusing on the open-access CXR image datasets. The most relevant publicly available CXR datasets are as follows:

1. **Indiana** is a publicly available dataset collected by Demner-Fushman et al. [38]. It has 7470 CXR images (frontal and lateral) and 3955 associated reports, collected from different hospitals and offered to the University of Indiana School of Medicine. The CXR images in this dataset represent several diseases such as pulmonary edema, opacity, cardiac hypertrophy, pleural effusion.
2. **ChestX-ray8** [39] is collected between 1992 and 2015. It contains 108,948 posterior images, with 24,636 containing one or more anomalies, and the remaining 84,312 images representing normal cases. The images belong to 32,717 patients. The dataset has labels that refer to eight diseases (pneumothorax, cardiomegaly, effusion, atelectasis, mass, pneumonia, infiltration, and nodule), where every image can be multi-labeled. The labels are text-mined from the associated radiological reports using NLP (natural language processing) algorithms.
3. **ChestX-ray14** [39] is a dataset of images extracted from the PACS (Picture Archiving and Communication Systems) databases. It is an upgraded version of ChestX-ray8 dataset with six more common chest abnormalities (hernia, fibrosis, pleural thickening, consolidation, emphysema, and edema). ChestX-ray14 has 112,120 frontal view CXR images (51,708 images contain one or multiple abnormalities and the remaining 60,412 images do not include any of the 14 abnormalities) belonging to 30,805 unique patients. ChestX-ray14 was also labeled using NLP techniques. Examples of CXR images from ChestX-ray14 are depicted in Figure 2.

4. **KIT** [40] is a tuberculosis dataset created by the Korea Association of Tuberculosis. It contains 10,848 DICOM images collected between 1962 and 2013, including 7200 normal cases and 3828 with TB.
5. **Montgomery** [41] is a dataset collected in collaboration with the US Department of Health and Human Services and Montgomery County. It has 138 frontal CXR images (80 normal and 58 with TB). The images are provided by Montgomery County’s Tuberculosis screening program.
6. **Japanese Society of Radiological Technology (JSRT)** [42] is a public dataset collected by the JSRT (Japanese Society of Radiological Technology) in collaboration with the JRS (Japanese Radiological Society) in 1998 from 13 institutions in Japan and one in the United States. It contains 247 postero-anterior CXR images, including 154 with nodule (100 CXR with malignant nodules and 54 with benign nodules) and 93 non-nodule high-resolution CXR images. JSRT has metadata such as diagnosis (malignant/benign), gender, age, and location of nodules [43].
7. **Shenzhen** [41] is composed of 662 CXR images, including 336 images showing TB and 326 images for normal cases. These CXR images were all captured in one month, and they include pediatric CXR. The Shenzhen dataset was collected in collaboration between Shenzhen No. 3 People’s Hospital and Guangdong Medical College in China.
8. **CheXpert** [44] is a large Public dataset of CXR images composed of 224,316 images acquired from 65,240 patients. It contains 14 common chest abnormalities, and it was collected from the Hospital of Stanford between 2002 and 2017. Each image in CheXpert dataset was labeled for the presence of 14 abnormality as negative, positive, or uncertain based on an automated rule-based labeller to extract the observations of experts from the free text radiology reports. Samples of CXR images from CheXpert are shown in Figure 3.

9. **Padchest (Pathology Detection in chest radiographs)** [45] is one of the biggest and most labeled public datasets, with 168,861 CXR images acquired from 67,000 patients from San Juan’s Hospital, Spain between 2009 and 2017. A total of 18 radiologists contributed in reporting Padchest dataset.
10. **PLCO** [46] is a large dataset with 185,241 CXR images of prostate, lung, colorectal and ovarian (PLCO) belonging to 56,071 patients (men and women). The PLCO dataset was collected in the context of investigating the impact of screening on cancer-related mortality and secondary endpoints in people aged between 55 and 74 years. It was created under the sponsorship of the NCI (National Cancer Institute).
11. **MIMIC-CXR** [47] is a collection of 377,110 CXR images corresponding to 227,835 patients. It is considered as one of the largest open-access datasets of chest radiographs with free text radiology reports. It has data of 14 chest abnormalities. It was performed between 2011 and 2016 at the Beth Israel Deaconess Medical Center (Boston, MA, USA).
12. **VinDr-CXR** [48] is a public CXR dataset with radiologist-generated annotations. It consists of 18,000 CXR images that come with the location and the classification of the chest diseases. This dataset was collected from two of the biggest hospitals in Vietnam that are Hospital H108 and the HMUH (Hanoi Medical University Hospital) [49]. Figure 4 shows CXR samples from VinDr-CXR dataset.

13. **Pediatric-CXR** [50] is collected from Guangzhou Women and Children’s Medical Center, China. It is composed of 5856 X-ray images (1583 normal cases and 4273 with pneumonia) of pediatric patients (one to five years) with different resolutions.
14. **The RSNA Pneumonia Detection Challenge dataset (RSNA-Pneumonia-CXR)** is collected by the RSNA (Radiological Society of North America) and the STR (Society of Thoracic Radiology) and published for a challenge [51]. It has 30,000 CXR images, of which 15,000 CXR are diagnosed with pneumonia or similar diseases such as infiltration and consolidation. Images in RSNA-Pneumonia-CXR dataset are all acquired from ChestX-ray14 dataset.
15. **COVIDx CXR-3** is a public benchmarking dataset that comprises a total of 30,386 CXR images from 17,026 patients. Images in COVIDx CXR-3 repository are collected by Pavlova et al. [52] from the following datasets:
  - **COVID Chest X-ray** [53], an open-access dataset obtained from public sources and by indirect collection from hospitals and physicians. It consists of 686 COVID-19 CXR images from 412 patients from 26 countries.
  - **COVID-19 Chest X-ray**, a COVID-19 dataset collected by Chung et al. [54] in collaboration with members from University of Waterloo in Canada. COVID-19 Chest X-ray dataset consists of 53 CXR COVID-19 images.
  - **Actualmed COVID chest X-ray**, a publicly available dataset of 217 CXR images, collected by Chung et al. [55] in collaboration with Actualmed and Jaume I University (UJI) in Castellón de la Plana, Spain.
  - **COVID-19-Radiography database**, created by a group of researchers at Qatar University in Qatar, and Dhaka University in Bangladesh, along with collaborators from Pakistan and Malaysia and a group of medical specialists [56]. It consists of 21,173 CXR images (3616 COVID-19, 6012 opacity, 1345 viral pneumonia and 10,200 normal).
  - **RSNA International COVID-19 Open Radiology Database (RICORD)** [57], created as a collaborative work between the RSNA and the STR. It comprises 998 CXR images with diagnostic labels (positive for COVID-19) belonging to 361 patients (aged 18 years or older) from four institutions across the world.
  - **BIMCV-COVID19+**, a large COVID-19 dataset That contains 3141 positive CXR images with radiology reports (pathologies, locations, and other details) and CT scan images [58]. It is published by the BIMCV (Valencian Region Medical Image Bank) in collaboration with the FISABIO (Foundation for the Promotion of Health and Biomedical Research of Valencia Region), and the Regional Ministry of Innovation, Universities, Science and Digital Society (Generalitat Valenciana).
  - **Stony Brook University COVID-19 Positive Cases (COVID-19-NY-SBU)**, a large collection of COVID-19 images from the “COVID-19 Data Commons and Analytic Environment” at the Renaissance School of Medicine, Stony Brook University [59]. COVID-19-NY-SBU dataset contains 562,376 images of different medical imaging modalities including X-rays acquired from 1384 patients.

**Table 1:** Publicly available CXR datasets.

Dataset	Ref.	Size	Classes	Collected/Sponsored by
Indiana ^*b*^	[38]	7470 images (512 × 512 pixels) 3996 patients	Multiple diseases including opacity, cardiomegaly, pleural effusion, and pulmonary edema	Indiana Network for Patient Care with various hospitals associated with the Indiana University School of Medicine
ChestX-ray8 ^*a*^	[39]	108,948 images (1024 × 1024 pixels) 30,805 patients	8 findings including pneumonia, atelectasis, mass, pneumothorax, infiltration, cardiomegaly, effusion, and nodule	From clinical PACS databases in the hospitals associated to NIHCC (National Institutes of Health Clinical Center)
ChestX-ray14 ^*a*^	[39]	112,120 images (1024 × 1024 pixels) 32,717 patients	14 findings including hernia, consolidation, emphysema edema, pleural thickening, pulmonary fibrosis, and others	From clinical PACS databases in the hospitals associated to NIHCC (National Institutes of Health Clinical Center)
KIT dataset ^*a*^	[40]	10,848 images	Normal and TB	The Korea Association of Tuberculosis between 1962 and 2013
Montgomery ^*b*^	[41,60]	138 images (4020 × 4892 pixels)	Normal and TB	Montgomery County Department of Health and Human Services
Shenzhen ^*b*^	[41]	662 images (3000 × 3000 pixels) 336 TB patients	Normal and TB	In collaboration with Shenzhen No. 3 People’s Hospital, Guangdong Medical College, Shenzhen, China
JSRT ^*b*^	[42,43]	247 images (2048 × 2048 pixels) 247 patients	Nodule and no nodule	Japanese Society of Radiological Technology
CheXpert ^*a*^	[44,61]	224,316 images 65,240 patients	14 findings including edema, cardiomegaly, lung opacity, lung lesion, consolidation, pneumonia, atelectasis, pneumothorax, and others	Stanford University Medical Center
Padchest ^*c*^	[45]	160,868 images 67,000 patients	Large number of findings	San Juan Hospital (Spain)
PLCO ^*a*^	[46]	185,241 images 56,071 patients	Prostate, lung, colorectal, and ovarian findings	The NCI (National Cancer Institute)
MIMIC-CXR ^*a*^	[47,62]	473,057 images (2544 × 3056 pixels) 63,478 patients	14 diseases (227,943 imaging studies)	MIT, Beth Israel Deaconess Medical Center (Boston, MA, USA)
VinDr-CXR ^*b*^	[48,49]	18,000 images	28 findings including TB, pneumonia, cardiomegaly, pleural effusion, lung opacity and others	The Hospital 108 (H108) and the HMUH (Hanoi Medical University Hospital)
Pediatric-CXR ^*b*^	[50,63]	5856 images	Normal, bacterial-pneumonia, viral-pneumonia	Guangzhou Women and Children’s Medical Center, China
RSNA-Pneumonia-CXR ^*b*^	[51]	15,000 images	Pneumonia, infiltration, and consolidation	The RSNA (Radiological Society of North America) and the STR (Society of Thoracic Radiology)

Note: ^*a*^ dataset annotated by an NLP algorithm, ^*b*^ dataset annotated by radiologists, ^*c*^ only 27% of CXR images were manually annotated by radiologists.

**Table 2:** Overview of publicly available COVID-19 CXR datasets.

Dataset	Ref.	Size	Classes	Collected/Sponsored by
COVIDx CXR-3	[52]	30,386 images	Positive and negative COVID-19	Pavlova et al. [52] by combining and modifying images from different COVID-19 datasets.
COVID Chest X-ray	[53]	686 images	Positive COVID-19	Cohen et al. [53] from public sources and by indirect collection from hospitals and physicians
COVID-19 Chest X-ray	[54]	53 images	Positive COVID-19	Chung et al. [54] in collaboration with members from University of Waterloo in Canada
Actualmed COVID chest X-ray	[55]	217 images	Positive COVID-19	Chung et al. [55] in collaboration with Actualmed and UJI (Jaume I University) in Castellón de la Plana, Spain
COVID-19-Radiography database	[56]	21,173 images	Normal, positive COVID-19, opacity, and viral pneumonia	A group of researchers at Qatar University and Dhaka University along with medical doctors and collaborators from Pakistan and Malaysia
RICORD	[57]	998 images	Positive COVID-19	The Radiological Society of North America and the Society of Thoracic Radiology
BIMCV-COVID19+	[58]	3141 images	Positive COVID-19, pneumonia, alveolar, and interstitial	The BIMCV (Valencian Region Medical Image Bank) in collaboration with the FISABIO (Foundation for the Promotion of Health and Biomedical Research of Valencia Region), and the Regional Ministry of Innovation, Universities, Science and Digital Society (Generalitat Valenciana)
COVID-19-NY-SBU	[59]	4118 images	Positive COVID-19	The Renaissance School of Medicine and Department of Biomedical Informatics at Stony Brook University

## 3. Image Preprocessing Techniques

Preprocessing of X-ray images is the operation that consists of improving their quality by converting them from their original form into a much more usable and informative form. Most of CXR images are produced in DICOM (Digital Imaging and Communications in Medicine) format with large set of metadata, which makes it challenging to understand by experts outside the field of radiology [47]. In other areas such as computer vision, DICOM images are usually stored in PNG or JPG formats using specific algorithms. These algorithms allow the compression without losing important information in the images. This process has two main steps, first is to de-identify the information of patients (privacy protection). Second is to convert DICOM images into PNG, JPEG, or other formats. Normal X-ray images have dimensions of 3000 × 2000 pixels, which requires high computational resources if used in their original size. Therefore, radiological images must be resized without losing the essential information they contain. Most of the datasets have resized images, such as Indiana dataset, which has CXR images resized to 512 × 512 pixels [38] and ChestX-ray dataset that has resized images with a dimension of 1024 × 1024 pixels [39].

Datasets are most of the time imbalanced or contain low-quality images, which usually contain noise and unwanted parts. In the process of developing a CAD system, the image preprocessing techniques play a crucial rule in enhancing and improving the quality of images. They help to remove the irrelevant data, to extract the meaningful information, and to make the ROI clearer. These techniques improve the performance of CAD systems and reduce their error rate. Preprocessing techniques applied on CXR images, consist of several methods including augmentation, enhancement, segmentation, and bone suppression.

### 3.1. Augmentation

Training a Deep Convolutional Neural Network (DCNN) on an imbalanced dataset mostly leads to overfitting, makes the model unable to generalize to novel samples and does not provide the desired results. To cope with this situation, many transformations can be employed by position-based augmentation (cropping, rotating, scaling, flipping, padding, elastic deformations) and color-based augmentation techniques (hue, brightness, contrast) to increase the number of samples in the dataset by making slight adjustments to existing images. Table 3 gives an overview of data-augmentation techniques applied to CXR images.

Ait Nasser and Akhloufi [64] used different data augmentation techniques, including a rotation of −15 to 15 degrees, a translation of 20% in four directions, a shear of 70 to 100, and a random flip, which resulted in a total of 84,204 CXR images. The increased data improved the performance of their proposed model. Nayak et al. [65] increased the number of CXR samples by applying different techniques such as rotation, scaling, horizontal flipping, and adding Gaussian noise with a variance between 0 and 0.25. These data-augmentation techniques resulted in five times more CXR samples than the original training set of images.

Over the past few years, generating artificial CXR images using generative adversarial networks (GANs) was of great interest to the research community, as it deals with the privacy concerns of patients data. GANs that have been introduced by Goodfellow et al. [66], were employed to increase the size of existing CXR datasets by creating new artificial images. GANs have two deep neural networks that are: the generator, which creates samples that are as realistic as possible to the original images, and the discriminator, which distinguishes original images from newly generated images. Venu et al. [67] employed GANs for data-augmentation using Pediatric-CXR dataset [63]. Most of the images in this dataset belong to one class (3875 Pneumonia, 1341 normal). This experiment consisted of increasing the number of normal CXR images using deep convolution adversarial networks (DC-GAN). After training the DC-GAN model, high quality CXR-like images were generated. Chuqucusma et al. [68] produced realistic lung nodule images by using unsupervised DC-GAN. The quality of generated nodules were evaluated by presenting Turing tests to two radiologists. Madani et al. [69] used DC-GAN to augment the original dataset by generating more CXR, and then trained a DCNN for a classification of abnormalities. The model showed high performance in classification. Data-augmentation techniques proved its efficiency on the performance of different models and systems for the detection and the classification of chest diseases using CXR images. Using DC-GAN, Albahli et al. [70] generated new synthetic CXR images to deal with the data imbalance problem. They trained several DL models with and without data augmentation. The results obtained show that data augmentation using DC-GAN improves the accuracy (ACC) by 5%.

**Table 3:** Overview of different data-augmentation techniques for CXR images.

Ref.	Dataset	Technique
[64]	Consolidated dataset of 26,316 CXR images from VinDr-CXR and CheXpert datasets	Rotation (−15 to 15 degrees), four directions translation (20%), shear (70 to 100), and a random flip
[65]	703 CXR images from ChestX-ray8 and COVID Chest X-ray and	Rotation, scaling, horizontal flipping, Gaussian noise (variance between 0 and 0.25)
[67]	1341 normal CXR images from Pediatric-CXR	DC-GAN
[68]	ChestX-ray14	Unsupervised DC-GAN
[69]	4110 CXR images from ChestX-ray14 and PLCO	DC-GAN
[70]	91,324 CXR from CheXpert	DC-GAN

### 3.2. Enhancement

**Figure 5:**
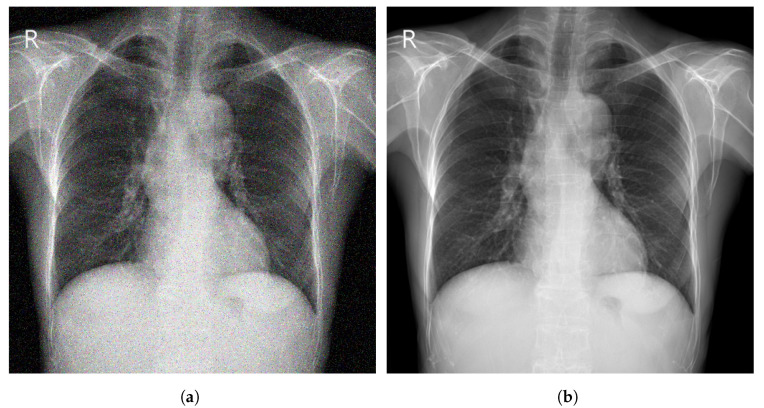
(**a**) Noisy CXR image from a low quality version of CheXpert dataset [44]; (**b**) Enhanced CXR image.

Image enhancement techniques are generally used to improve the information interpretability in images. For CXR images, these techniques are used to provide a better image quality to human readers (radiologists) as well as to automated systems [71]. To improve the quality of a CXR image, multiple parameters can be considered (contrast, brightness, noise suppression, edge of features, and sharpness of edges) using different methods including histogram equalization (HE) [72], high and low pass filtering [73], and unsharp masking [74]. Figure 5 depicts an example of enhancement applied to a CXR image.

Aashiq et al. [75] used a gabor filter that represents a combination of gaussian and a sinusoidal term to enhance CXR images. Munadi et al. [76] employed three techniques (CLAHE (contrast-limited adaptive histogram equalization), high frequency emphasis filtering, and unsharp masking) to enhance TB images from Shenzhen dataset. A transfer learning technique was used to train two DCNN models to detect TB using enhanced images. Tawsifur et al. [77] explored the effect of various enhancement techniques on the performance of DCNN models for COVID-19 detection using CXR images. The used enhancement techniques include, HE, CLAHE, image invert, gamma correction, and Balance Contrast Enhancement Technique (BCET). Nefoussi et al. [78] enhanced CXR pneumonia images using several preprocessing techniques such as unsharp mask, CLAHE, and HE. Zhou et al. [79] proposed an enhancement method for CXR images. They applied techniques to tune the brightness and contrast of the input image and obtain an enhanced image in the output. This method is based on a parameter called gamma. Genc et al. [80] proposed three preprocessing approach, including two enhancement techniques (HE and CLAHE) using CXR images from ChestX-ray14 dataset. Koonsanit et al. [81] used an enhancement technique called N-CLAHE which combines the normalization function and the CLAHE method to increase the quality of CXR images. N-CLAHE provided the highest contrast resolution when compared with other techniques such as CLAHE, HE, and unsharp masking. Kushol et al. [82] performed a contrast enhancement method for CXR images from six datasets including Montgomery, ChestX-ray14 and Shenzhen. The proposed method used two techniques (top hat and bottom hat transform) to enhance the quality of CXR images based on features extraction and background equalization.

Most studies showed that using enhancement techniques improves the performance of automatic systems for feature extraction and disease detection [77,79,80]. An overview of enhancement techniques applied to CXR images is shown in Table 4.

### 3.3. Segmentation

**Figure 6:**
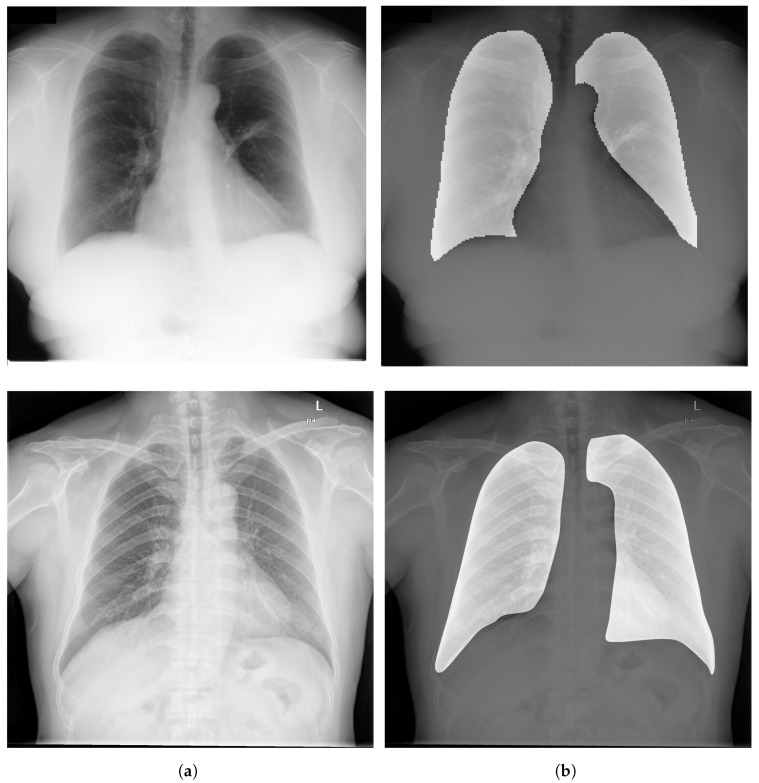
(**a**) Examples of CXR images from CheXpert dataset [44]; (**b**) Samples of ROIs overlaid on CXR images.

Image Segmentation has a critical rule in image preprocessing techniques. It is usually necessary to divide a visual image into fragments. For CXR images, this technique allows segmenting the thoracic image into areas in order to extract the ROI. Figure 6 depicts examples of ROIs overlaid on CXR images.

CXR image segmentation was implemented in several studies and research, and it proved its efficiency in the whole process of detection and classification of chest diseases. For instance, Kumarasinghe et al. [83] used U-Net model, that is among the most efficient methods to perform segmentation of the CXR images. The generated images were eventually used for a classification of pneumonia and COVID-19 cases. Gu et al. [84] used a fully convolutional networks (FCN) model to perform lung field segmentation and isolate the anatomical ROI using CXR images. Sogancioglu et al. [85] explored the detection of cardiomegaly on CXR images from ChestX-ray14 dataset, using two approaches (segmentation and classification). They proposed a standard U-Net architecture that segments the lungs and heart regions. Eslami et al. [86] proposed a DL model, namely pix2pix, for organ segmentation using JSRT dataset. The pix2pix model suppresses the bones and segments the organs within the chest area (heart and lungs) of CXR images. Ghali and Akhloufi [87] proposed a segmentation model called ARSeg for identification and segmentation of lung fields. Attention mechanism was employed to suppress irrelevant features in CXR images. The model was performed on images from three public datasets (Shenzhen, Montgomery, and JSRT). Dai et al. [88] proposed a SCAN (structure correcting adversarial network) to segment heart and lung regions in CXR images from JSRT dataset. Liu et al. [89] proposed an improved U-Net model to extract lung field features using CXR images from two datasets (Montgomery and JSRT).

Most cited papers in this section proved that using segmentation techniques for the extraction of ROI using DL models such as U-Net [90] and FCN [91] has high potential to improve the performance of DCNN models for chest disease detection and classification [83,86,89]. Table 5 shows an overview of segmentation techniques applied to CXR images.

### 3.4. Bone Suppression

**Figure 7:**
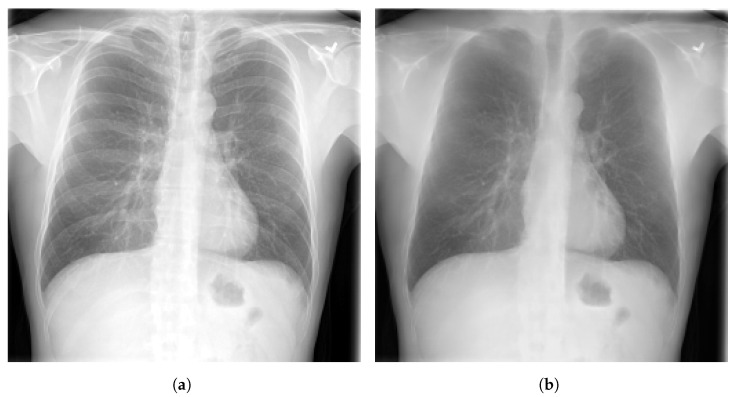
(**a**) CXR image from CheXpert dataset [44] before bone suppression; (**b**) CXR image after bone suppression.

Bone suppression is a technique that can be applied on CXR images. It is an important step in the process of lung segmentation and extraction of features from thoracic images. Bone suppression technique is based on removing the bones from the CXR images, as depicted in Figure 7. It helps to increase the visibility of obscure zones and to prevent the overlap of signs of diseases with ribs and clavicles. An overview of bone suppression techniques applied to CXR images is presented in Table 6.

Several studies have investigated this technique of bone suppression including, Matsubara et al. [92] who proposed a convolutional neural filter (CNF) that suppresses all bone components without loss of tissue information. Sato et al. [93] developed a bone suppression algorithm that is based on gradient differences in CXR images. Zarshenas et al. [94] presented a CNN model to separate the bone structures from soft tissue in different lung regions using a dataset of CXR radiographs with pulmonary nodules. Rajaraman et al. [95] proposed a DCNN model called DeBoNet that removes bones in CXR images. The generated images were used to detect COVID-19. Zhou et al. [96] introduced a conditional GAN model, for suppressing bone shadow without loosing contextual information. The model generated boneless images of high quality. Gordienko et al. [97] applied a DL method to detect lung cancer. They compared the performance of the model by applying it on CXR datasets with and without bones. Using CXR dataset of radiographs without clavicle and rib shadows, the model showed higher performance.

The papers cited in this section, showed the usefulness of the bone suppression technique in increasing the performance and the reliability of DCNN models for the detection of different diseases [92,93,94].

**Table 6:** Overview of different bone suppression techniques for CXR images.

Ref.	Dataset	Technique
[92]	ChestX-ray8 and JSRT	Convolutional neural filter
[93]	604 CXR images from a private dataset	Custom algorithm based on gradient differences in CXR images
[94]	118 CXR images with pulmonary nodules	Custom CNN model
[95]	3016 CXR images from BIMCV-COVID19+, ChestX-ray14, and RSNA-Pneumonia- CXR	DeBoNet
[96]	JSRT	Conditional GAN

### 3.5. Evaluation Metrics

Several metrics for evaluating the performance of CAD systems are available. The commonly used metrics in medical imaging analysis are given in the following:

- Accuracy (ACC), which determines the number of correct predictions out of all predictions.
  (1) $$ACC=\frac{TP+TN}{TP+FN+TN+FP}$$
- Precision (PRE), which determines number of correct positive predictions.
  (2) $$PRE=\frac{TP}{TP+FP}$$
- F1-score, which describes the harmonic mean of the recall and the precision.
  (3) $$F1\text{-}\mathrm{score}=\frac{2.TP}{2.TP+FP+FN}$$
- Sensitivity (SEN), also called recall, measures the ability to identify abnormal cases.
  (4) $$SEN=\frac{TP}{TP+FN}$$
- Specificity (SPE), which measures the ability of not reporting normal cases as abnormal.
  (5) $$SPE=\frac{TN}{TN+FP}$$
  where TP represents the true positive rate, FP represents the false positive rate, TN represents the true negative rate, and FN represents the false negative rate.
- Area under curve (AUC) which is one of the commonly used metrics in medical imaging analysis using CAD systems. AUC describes the performance of a proposed model based on its bad and good predictions.

Other metrics usually used for medical image segmentation are given in the following:

- Dice index, which is a function to measure the performance of the segmentation and the overlap similarity between image (A) and image (B).
  (6) $$\mathrm{Dice}\left(A,B\right)=2\frac{\left|A\cap B\right|}{\left|A|+|B\right|}$$
- Jaccard index, also known as IoU (intersection over union), is one of the most used metrics in segmentation. It is very similar to dice index as it evaluates the agreement between the ground truth (G) and the predicted segmentation (S).
  (7) $$\mathrm{Jaccard}\left(S,G\right)=\frac{\left|S\cap G\right|}{\left|S\cup G\right|}$$

## 4. Deep Learning for Chest Disease Detection Using CXR Images

Several CAD systems were developed to detect chest diseases using different techniques. Early diagnosis of thoracic conditions gives a chance to overcome the disease. Diseases such as TB, pneumonia, and COVID-19 become more serious and severe when they are at an advanced stage. In CXR images, three main types of abnormalities can be observed: (1) Texture abnormalities, which are distinguished by changes diffusing in the appearance and structure of the area. (2) Focal abnormalities, that occur as isolated density changes and (3) Abnormal form where the anomaly changes the outline of the normal morphology. A plethora of models and systems developed to deal with chest diseases using DL approaches are presented in this paper. We focused on DL algorithms applied for detecting different chest diseases using CXR images.

In this section, we review recently published DL approaches for binary and multi-disease detection, splitting them into sections on the basis of the disease detected (pneumonia, pulmonary nodule, TB, COVID-19, and multiple diseases). We selected the listed diseases based on their mortality and spread rates according to the WHO reports.

### 4.1. Pneumonia Detection

It is a challenging task for radiologists to detect pneumonia using CXR images. Pneumonia may appear as a benign abnormality and overlap with other diseases [98]. Multiple works were developed in the context to avoid misdiagnosing pneumonia, as shown in Table 7. For example, Ma and Lv [99] proposed a Swin transformer model for features extraction with a fully connected layer for classification of pneumonia in CXR images. The performance of the model was evaluated against DCNN models using two different datasets (Pediatric-CXR and ChestX-ray8). Image enhancement and data-augmentation techniques were applied, which improved the performance of the introduced model, achieving an ACC of 97.20% on Pediatric-CXR and 87.30% on ChestX-ray8. Singh et al. [100] proposed an attention mechanism-based DCNN model for the classification of CXR images into two classes (normal or pneumonia). ResNet50 with attention achieved the best results with an ACC of 95.73% using images from Pediatric-CXR dataset. Darapaneni et al. [101] implemented two DCNN model with transfer learning (ResNet-50 and Inception-V4) for a binary classification of pneumonia cases using CXR images from RSNA-Pneumonia-CXR dataset. The best performing model was Inception-V4 with a validation ACC of 94.00% overcoming ResNet-50 which achieved a validation ACC of 90.00%. Rajpurkar et al. [102] developed CheXNet model, which is composed of 121-layer convolutional network to detect and localize the lung areas that show the presence of pneumonia. ChestX-ray14 was used to train the model, which was fine-tuned by replacing the final fully connected layer with one that has a single output. A nonlinear sigmoid function was used as an activation function, and the weights were initialized with the weights from ImageNet. CheXNet model showed high performance, achieving an AUC of 76.80%. Kundu et al. [103] developed a CAD system based on transfer learning for a binary classification of pneumonia. Their proposed approach consists of training an ensemble learning of three different DCNN models (ResNet-18, GoogleNet, and DenseNet-121) with a weighted average ensemble technique and a five cross-validation strategy. Two publicly available datasets were used for this experimentation (Pediatric-CXR and RSNA-Pneumonia-CXR). The proposed model achieved an ACC of 98.81% on Pediatric-CXR and an ACC of 86.86% on RSNA-Pneumonia-CXR dataset.

Khoiriyah et al. [9] used 5856 CXR of the Pediatric-CXR dataset to classify images into two classes (binary classification) normal and pneumonia. They increased the training data by applying data-augmentation techniques. The generated data were used to train a customized CNN, achieving an ACC of 83.38% on detecting pneumonia. Zhang et al. [104] used a CAAD (confidence-aware anomaly detection) model to identify viral pneumonia from non-viral pneumonia. Two CXR datasets were used (X-viral and X-Covid) in this experiment. The first one has 5977 images with viral pneumonia and 37,393 images with non-viral pneumonia. The second dataset has 106 COVID-19 images and 107 healthy [104]. The two datasets were collected from 390 hospitals via a telemedicine platform of JF Healthcare [107]. The proposed CAAD model showed superior performance, achieving an AUC of 87.57% for viral pneumonia screening. Siddiqi [98] used an 18-layer DCNN algorithm for a classification of CXR images into pneumonia or normal. Pediatric-CXR dataset was used to accomplish this task. The model achieved a 94.39% ACC, a 99.00% SEN, and an 86.00% SPE. Sharma et al. [105] developed a DCNN model for the extraction of features and the detection of pneumonia using Pediatric-CXR dataset. They opted for data-augmentation techniques such as resize, flip, and rotation. The model was tested with and without dropout and data-augmentation. The results showed that the model performed better with dropout and data-augmentation, achieving an ACC of 90.00%. Kermany et al. [63] used the Pediatric-CXR dataset to detect pneumonia and to distinguish viral from bacterial pneumonia using the transfer learning technique. The model Inception-V3 achieved an ACC of 92.80% for the detection of pneumonia from normal cases, and an ACC of 90.70% for the identification of viral and bacterial pneumonia. Stephen et al. [106] proposed a DCNN model trained from scratch for the classification and detection of pneumonia using Pediatric-CRX dataset. Due to the small size of the dataset, different algorithms for data-augmentation were used to overcome overfitting and to reduce the generalization error. The model achieved high results with an ACC of 93.73%.

### 4.2. Pulmonary Nodule Detection

According to the WHO, lung cancer is one of the most dangerous diseases. It is the most frequent cancer in men and the third in women [108]. Lung cancer manifests as lung nodules. Early diagnosis of these nodules is extremely effective in treating lung cancer before it becomes dangerous.

To demonstrate the usefulness of DL-based systems to assist radiologists in detecting pulmonary nodules on medical images, several studies were carried out. DL algorithms proved high performances on different modalities of medical screening and specifically on X-ray radiographs. For instance, to compare the performance of radiologists for the detection of malignant pulmonary nodules with and without assistance of a DL based CNN system, Sim et al. [109] proved in a multi-centre study that the performance of 12 radiologists was improved with more than 5% in terms of SEN after being assisted by a DL system. The performance of radiologists increased from 65.10% to 70.30% when they used the proposed DCNN software. Cha et al. [6] trained a model based on a ResNet architecture for detecting operable lung cancer. In this experiment, the authors used a dataset composed of 17,211 CXR images, augmented to 600,000 using different techniques such as cropping, resize and rotation. The model achieved a SEN of 76.80% and an AUC of 73.20% outperforming six radiologists in the detection of lung cancer. Thamilarasi et al. [110] used a DL approach for automatic classification of lung nodules into normal or abnormal. The proposed architecture consists of a custom DCNN model trained and tested on 180 segmented CXR images (90 nodules and 90 non-nodule images) acquired from JSRT dataset. Data-augmentation techniques were performed to increase the number of CXR samples and to avoid overfitting. The results showed that the introduced model achieved a high ACC of 86.67%. Bush [111] proposed ResNet-50 model used with transfer learning for the classification of CXR images from JSRT dataset into non-nodule, benign, or malignant nodule. The model achieved 92.00% for SEN and 86.00% for SPE. Pesce et al. [112] used a very large dataset collected from the historical archives of Guy’s and St. Thomas NHS foundation (contains 745,479 CXR images) to train a convolutional neural network with attention feedback to detect lung nodules. The used model obtained a 92.00% PRE, a 78.00% SEN, an 85.00% ACC and an 85.00% F1-score. Schultheiss et al. [37] trained a DCNN RetinaNet model using a dataset of 411 CXR. The performance of the model was compared with that of two radiologists in detecting pulmonary nodules on segmented images. The RetinaNet model achieved an AUC of 87.00% outperforming the two radiologists. Wang et al. [113] experienced the potential of DL in detecting pneumoconiosis by training the model Inception-V3 with fine-tuning on a dataset composed of 1881 chest X-ray images (958 Normal and 923 diagnosed with pneumoconiosis) obtained from the PACS database at Pekin University Third Hospital. The Inception-V3 model achieved an AUC of 87.80%, outperforming two radiologists who achieved an AUC of 66.80% and 77.20%. Xuechen et al. [114] introduced a DenseNet-based architecture to detect lung nodules. The proposed model was trained using CXR Images from JSRT dataset after applying preprocessing techniques such as lung area segmentation and bone suppression. An extraction of patches was performed for each pixel in the segmented lung area. A full feature fusion technique was then applied to perform a combination of extracted features. The proposed DCNN model achieved a high ACC of 99.00%, outperforming the average predictions of radiologists. Kim et al. [115] used two DCNN models that are Mask R-CNN and RetinaNet to validate the effects of using different sizes of CXR images (256, 448, 896, 1344, and 1792). A dataset of 2088 abnormal (nodule/mass) and 352 normal CXR radiographs collected from CheXpert was used. A total of 896 and 1344 pixels in Mask R-CNN and 896 in RetinaNet were the optimal sizes. The two models showed high performance in terms of SEN, achieving 95.60%. Table 8 represents an overview of presented works for pulmonary nodule classification.

### 4.3. Tuberculosis Detection

According to the WHO, TB is ranked on the top 10 diseases leading to death. TB is ranking as the second infectious disease leading to death after COVID-19 and above HIV/AIDS. In 2020, around 10 million people suffered from TB (1.1 million children). It killed a total of 1.4 million people in 2019 and 1.5 million people in 2020. TB is caused by the bacillus mycobacterium TB, which spreads when people who are sick with TB expel bacteria into the air (by coughing or sneezing). The disease typically affects the lungs [10].

An early diagnosis of TB saved an estimation of 66 million lives between 2000 and 2020. The variety of manifestations of pulmonary TB on CXR images makes the detection a challenging task. DL proved its high efficiency in the detection and the classification of TB. Ahmed et al. [116] proposed an approach to overcome TB detection problem using an efficient DL network named TBXNet. TBXNet is implemented using five dual convolution blocks with different filter sizes of 32, 64, 128, 256 and 512, respectively. The dual convolution blocks are merged with a pre-trained layer in the fusion layer of the architecture. Moreover, the pre-trained layer is used to transfer pre-trained knowledge into the fusion layer. The proposed TBXNet obtained an ACC of 99.17%. All experiments are performed using image data acquired from different sources (Montgomery, a labeled dataset created by different institutes under the ministry of health of the Republic of Belarus and a labeled dataset, that was acquired by the kaggle public available repository). Tianhao and Zhenming [117] proposed an automated TB detection model called VGG16-CoordAttention. The proposed approach involves implementing a coordinated attention mechanism to the architecture of VGG-16. A comparative analysis of over five different deep learning models including Version-Transformer (VIT), VGG-16, ResNet-50, MobileNet-V2 and the proposed model to determine which is the most effective type model to detect TB. All models were frozen for 30 epochs and unfrozen for the rest of the 90 epochs during the training process. All experiments carried out were performed using the Shenzhen dataset. Results show the effectiveness of the attention mechanism with an ACC of 92.73%, a PRE of 92.73%, an F1-score of 92.82% and an AUC of 97.71%. Eman et al. [118] introduced a DCNN model (ConvNet) trained from scratch to automatically detect TB on CXR images. Furthermore, a transfer learning techniques with five different pre-trained models (VGG-16, VGG-19, Inception-V3, ResNet-50 and, Xception) was used to evaluate the performance of each model with the proposed technique. CXR images from Montgomery dataset and Shenzhen datasets were used for this experiment. The proposed DCNN architecture (ConvNet) achieved an 88.0% PRE, an 87.0% SEN, an 87.0% F1-score, an 87.0% ACC and an AUC of 87.0%. Lakhani et al. [119] employed an ensemble learning method using two DCNN models (AlexNet and GoogleNet) to classify CXR images from Shenzhen dataset into TB or normal. Different preprocessing techniques such as image contrast enhancement and rotations were applied to improve the ACC in cases where classification is uncertain and ambiguous. The used model achieved 99.00% in terms of AUC. Hwang et al. [60] used a DCNN AlexNet model with transfer learning to overcome the challenges of training from scratch and to improve the performance of the system. Three datasets were used in this study (KIT, Montgomery, and Shenzhen). The model achieved high results, achieving an ACC of 90.30% and an AUC of 96.40%. Seelwan et al. [11] developed a DCNN model using two CXR datasets. They acquired TB images from Shenzhen and non-TB images from National Institute of Health Clinical Centers (NIH). A set of tests were applied and the DCNN model obtained high results (AUC of 98.45%, SEN of 72.00%, and SPE of 82.00%). Tawsifur et al. [120] created a dataset of 3500 TB and 3500 normal CXR images using several public datasets such as Montgomery and Shenzhen datasets. They used nine pretrained DCNN models (ResNet-101, ResNet-50, ResNet-18, SqueezeNet, Inception-V3, CheXNet, VGG-19, DenseNet-201, and MobileNet) and transfer learning. DenseNet-201 achieved the highest results with an ACC of 98.60%, a PRE of 98.57%, a SEN of 98.56%, F1-score of 98.56%, and an SPE of 98.54% for the segmented lung images. Subhrajit et al. [121] proposed a framework that combines multiple DCNN models pre-trained on ImageNet dataset including DenseNet-121, VGG-19 and ResNet-50. The type-1 Sugeno fuzzy integral based ensemble technique was used to average the predictions of used models. The proposed framework achieved a high classification ACC of 99.75% outperforming the state-of-the-art works. All experiments were performed using the CXR dataset created by Tawsifur [120]. Hooda et al. [122] presented a DL approach to classify CXR images into two classes (normal and TB) using Montgomery and Shenzhen datasets. They used a DCNN model with three different optimizers. The best results were obtained using Adam as an optimizer, with an 82.09% ACC. Nguyen et al. [123] used a pre-trained DenseNet model for the classification of CXR images into normal and TB. The images used in this work are acquired from two datasets (Shenzhen and Montgomery). Using an improved transfer learning method with DenseNet-121, the model obtained an AUC of 99.00% on Shenzhen dataset and an AUC of 84.00% on Montgomery dataset. Lopes et al. [124] employed an ensemble learning approach using different pre-trained DCNN models as features extractors to classify CXR images into TB and normal. To evaluate the models, they used two public datasets (Shenzhen and Montgomery). The best model achieved an ACC of 80.00%. Meraj et al. [125] used four DCNN models to evaluate their limits using the ACC and the AUC metrics for classification of TB. The used models are: GoogleNet, ResNet-50, VGG-19, and VGG-16. Two public datasets were used in this study (Montgomery and Shenzhen). The tests proved that VGG-16 model achieved the highest scores with an ACC of 86.74% and AUC of 92.00% outperforming the three other models. Abbas et al. [126] opted for an approach based on a class decomposition method to deal with the complexity of data distribution, and consequently to increase the performance of ImageNet pre-trained models using transfer learning techniques. Several DCNN models were trained with and without class decomposition (e.g., AlexNet, GoogleNet, ResNet). This approach achieved high ACC for TB detection on the publicly available JSRT dataset [43], achieving 99.80% in terms of ACC.

In Table 9 we summarize the deep learning methods used for TB detection, the used datasets, the evaluation metrics, and the obtained results.

### 4.4. COVID-19 Detection

In late 2019, COVID-19 first appeared in Wuhan, China. It was officially announced as a pandemic by the WHO in early 2020 due to its rapid spread and dangerous effects on humans. The detection of COVID-19 virus in humans is usually performed using different clinical techniques that are costly and time-consuming. To deal with this challenge, CXR images were used for the detection of COVID-19. Millions of people have died because of this pandemic, and the number is still increasing on a daily basis. DL techniques using CXR images are helpful in detecting and monitoring the effects of COVID-19 on lung tissue. The use of DL algorithms for COVID-19 detection and classification was a challenge at the beginning of the pandemic because of lack of CXR for positive images. The challenge for the research community was to create open access CXR datasets with COVID-19 cases to advance the research and develop solutions to help overcome this pandemic. Several techniques and models of DL were conducted, and they showed high performances that ranged from 89.00% to 98.00% for multi-class classification and from 90.00% to 99.00% for two and three-class classification. In Table 10 we present an overview of obtained results for COVID-19 detection. Islam et al. [127] proposed a DCNN model with five convolutional blocks to autonomously diagnose COVID-19 disease in CXR images. Each block is composed of multiple layers, and every layer has a ReLU (rectified linear unit) activation function. In the third and fourth blocks a dropout layer was implemented to reduce the over-fitting issue. Two FCLs (fully connected layers) were used in this study. The first FCL was employed with the dropout layer, and the last FCL was used with the softmax classifier. This architecture achieved 97.00% SPE, 96.30% ACC, 96.00% PRE, 96.00% SEN, and 96.00% F1-score. A dataset of 10,293 CXR images was used in this experimentation (2875 COVID-19, 4200 pneumonia, and 3218 normal CXR images). It was collected from COVID Chest X-ray dataset, Pediatric-CXR dataset and a kaggle repository [128]. Alqahtani et al. [129] proposed a DL approach that uses readily available CXR images to identify COVID-19 cases. They employed an Inception-V4 model with transfer learning for the automatic detection of COVID-19 using CXR images. A total of 1504 chest images (504 images of COVID-19 cases, and 1000 normal cases) were used in this study collected from Pediatric-CXR and COVID-19 Chest X-ray datasets. The proposed model detected COVID-19 infection with an overall ACC of 99.63%.

Malathy et al. [130] presented a DL model called CovMnet to classify CXR images into normal and COVID-19. The layers in CovMnet include a convolution layer along with a ReLU activation function and a MaxPooling layer. The output of the last convolutional layer in the architecture is flattened and fit to fully connected neurons of four dense layers, activation layer and Dropout. Experiments are carried out for deep feature extraction, fine-tuning of CNNs (convolutional neural networks) hyperparameters, and end-to-end training of four variants of the proposed CovMnet model. The introduced CovMnet achieved a high ACC of 97.40%. All experiments were performed using CXR images from Pediatric-CXR dataset. Şengür et al. [131] employed three approaches for detecting COVID-19. Two of them were based on transfer learning and fine-tuning approaches (deep feature extraction). The third was an end-to-end new DCNN model. A dataset composed of 200 normal and 180 COVID-19 CXR images from COVID Chest X-ray and Pediatric-CXR datasets was used for the experiment. All images in this dataset were labeled by specialists. Techniques of data-augmentation were used for both fine-tuning and end-to-end training. Fine-tuning of five pretrained models (VGG-16, VGG-19, ResNet-18, ResNet-50, and ResNet-101) was performed to accurately extract deep features. The best results were obtained by ResNet-50, achieving 95.79% for ACC, 94.00% for SEN and 97.78% for SPE. Brunese et al. [132] adopted a three-step approach to detect pneumonia and COVID-19. The first step is based on the detection of the presence of pneumonia on CXR images. The second step is to distinguish between the two diseases (pneumonia and COVID-19). The last step focuses more on the localization of areas that show the presence of COVID-19. A dataset of 6523 CXR images of the thorax acquired from different sources were used in this study (ChestX-ray14 dataset and COVID Chest X-ray dataset). The VGG-16 model achieved a mean ACC of 97.00%. Ahsan et al. [133] proposed a DL model to detect COVID-19 patients on two different datasets, one of which is composed of CT scan images and the second contains 400 CXR images (200 normal and 200 COVID-19). They used eight DL models with modifications on the hyperparameters. For CXR images, the model that was able to achieve the best results is NasNetMobile, overcoming seven other models by attaining an ACC of 93.94%. Apostolopoulos et al. [134] trained a DCNN models to detect COVID-19 from common pneumonia. Different public repositories were used to collect images. The authors collected images from COVID Chest X-ray dataset, Pediatric-CXR dataset and a medical repository available on kaggle [135]. Five models were trained on the dataset using transfer learning parameters (Inception, MobileNet-V2, VGG-19, Inception, and ResNet-v2). MobileNet-V2 performed better than other models, achieving an ACC of 96.78%, an SEN of 98.66% and an SPE of 96.46%. Nguyen et al. [136] trained the DenseNet-121 DCNN model on 21,165 CXR images from different datasets including COVID-19-Radiography, RICORD, BIMCV-COVID19+, and Pediatric-CXR. After applying a HE technique and a geometric data-augmentation technique, the proposed model achieved a 97% ACC for binary classification of COVID-19 cases. Bekhet et al. [137] proposed a DCNN model for COVID-19 detection. The model was trained on COVID Chest X-ray public dataset [53] which contains nine types of pneumonia (e.g., SARSr-CoV-1, SARSr-CoV-2, and MERS-CoV). The used dataset contains 316 CXR images. Data-augmentation techniques were performed to reduce the effect of overfitting [138]. The proposed model achieved an ACC of 96.00%. Sethy et al. [139] investigated 13 different DCNN models (AlexNet, ShuffleNet, Inception-V3, InceptionResNet-V2, Resnet-18, Resnet-50, Densenet-201, Resnet-101, MobileNet-V2, VGG-19, VGG-16, Xception, and GoogleNet) using transfer learning technique. VGG-19 obtained the best ACC (99.81%) using a CXR dataset composed of 700 images from COVIDx CXR-3 (350 COVID-19 and 350 normal) outperforming 39 ML approaches. Chetoui et al. [140] proposed an explainable approach for binary and multi-disease classification. Nine datasets were merged to train the fine-tuned DCNN (EfficientNet-B5). An explainable approach was performed using the Grad-CAM (Gradient-weighted Class Activation Mapping) algorithm [141] to output the heatmaps. More than 3200 COVID-19 CXR images from COVIDx repository and Perdiatric-CXR dataset were used in this experimentation. EfficientNet-B5 obtained an average AUC of 98.00% for binary classification of COVID-19 and an average AUC of 97.00% for multi-classification of pneumonia, COVID-19 and normal. Hemdan et al. [142] proposed COVIDX-Net framework automatic detection of COVID-19. The framework has seven different architectures of DCNN models that are InceptionResNet-V2, DenseNet-201, Inception-V3, ResNet-V2, Xception, VGG-19, and MobileNet-V2. COVID Chest X-ray dataset was employed to train the models. VGG-19 and the DenseNet models obtained the best results, achieving respectively an F1-score of 89.00% and 91.00% for Normal and COVID-19. Khan et al. [143] used three fine-tuned DCNN models (EfficientNet-B1, MobileNet-V2, and NasNetMobile) with data-augmentation to detect and classify CXR images into four classes (normal, COVID-19, pneumonia, and lung opacity). EfficientNet-B1 achieved an ACC of 96.13%, outperforming the other models. A collection of 21,165 CXR images collected from different public datasets including BIMCV-COVID19+, Pediatric-CXR, RSNA-Pneumonia-CXR, and COVID-19-Radiography was used in this study. Wang et al. [144] proposed a custom ResNet model used with the multi-head self attention mechanism (MHSA-ResNet) for the classification of CXR images into three classes (normal, pneumonia, and COVID-19). A GLCM algorithm was used to extract the texture features from the CXR images. A custom dataset of 5173 CXR images from COVIDx CXR-3 dataset were used in this study. The model obtained an ACC of 95.52% and a PRE of 96.02%.

**Table 10:** Summary of different DL architectures for COVID-19 detection.

Ref.	Dataset	Model	Results
[127]	CXR images collected from COVID Chest X-ray dataset, Pediatric-CXR dataset and a kaggle repository [128]	Custom DCNN model with five convolutional blocks	ACC = 96.30% SEN = 96.00% PRE = 96.00% SPE = 97.00% F1-score = 96.00%
[129]	Custom dataset of 1504 CXR images (504 for COVID-19, and 1000 for normal cases) collected from Pediatric-CXR and COVID-19 Chest X-ray	Inception-V4 with transfer learning	ACC = 99.63%
[130]	648 CXR images acquired from Pediatric-CXR dataset	Custom DCNN model (CovMnet)	ACC = 97.40%
[131]	Custom dataset consists of 180 COVID-19 and 200 Normal CXR from COVID Chest X-ray and Pediatric-CXR datasets	ResNet-50	ACC = 95.79% SEN = 94.00% SPE = 97.78%
[132]	Custom dataset contains, 6523 CXR images acquired from ChestX-ray14 dataset and COVID Chest X-ray datasets	Transfer learning with VGG-16	ACC = 97.00%
[133]	400 CXR collected from Pediatric-CXR dataset	NasNetMobile	ACC = 93.94%
[134]	Custom dataset by merging images from three datasets (COVID Chest X-ray dataset, Pediatric-CXR dataset and a medical repository available on kaggle [135])	Transfer learning with MobileNet-V2	ACC = 96.78% SEN = 98.66% SPE = 96.46%
[136]	COVID-19-Radiography, Pediatric-CXR, BIMCV-COVID19+, and RICORD	DenseNet-121	ACC = 97.00%
[137]	COVID Chest X-ray	Custom DCNN model	ACC = 96.00%
[139]	COVIDx CXR-3	VGG-19	ACC = 99.81%
[140]	Custom dataset with more than 3200 COVID-19 CXR images collected from COVIDx CXR-3 repository, Perdiatric-CXR, Montgomery, Shenzhen, and ChestX-ray14	EfficientNet-B5	AUC = 98.00%
[142]	COVIDx CXR-3	VGG-19	F1-score = 91.00%
[143]	Custom dataset with 21,165 CXR images from BIMCV-COVID19+, Pediatric-CXR, RSNA-Pneumonia-CXR, and COVID-19-Radiography	EfficientNet-B1	ACC = 96.13%
[144]	Custom dataset with 5173 CXR images from COVIDx CXR-3	Custom DCNN model (MHSA-ResNet)	ACC = 95.52% PRE = 96.02%

### 4.5. Multiple Disease Detection

In some cases, a patient may suffer from more than one disease at the same time, which can put his life at higher risk. It may be difficult for radiologists to detect more than a pathology using CXR images due to the similarities between the signs of diseases. In such a situation, more details and more exams may be needed. To deal with this challenge, several DL systems were carried out using different algorithms. For instance, Majdi et al. [145] proposed a fine-tuned DenseNet-121 to classify CXR images into pulmonary nodules and cardiomegaly diseases. Images from CheXpert dataset were used for the experiment. The model obtained an AUC of 73.00% for pulmonary nodule detection and 92.00% for cardiomegaly detection. Bar et al. [146] employed a DL technique for the detection of pleural effusion, cardiomegaly, and normal versus abnormal disease by using a combination of features extracted by the DCNN model and the low-level features. Preprocessing techniques were applied on the used dataset that contains 93 CXR images collected from Sheba Medical Center. They attained an AUC of 93.00% for pleural effusion, 89.00% for cardiomegaly, and 79.00% for normal versus abnormal cases. Cicero et al. [147] used GoogleNet model to classify frontal chest radiograph images into normal, consolidation, cardiomegaly, pulmonary edema, pneumothorax, and pleural effusion. GoogleNet achieved an AUC score of 86.80% for edema, 96.20% for plural effusion, 86.10% for pneumothorax, 96.40% for normal, 87.50% for cardiomegaly and 85.00% for consolidation. The study proved that the DCNN model can achieve high performance even if trained with modest-sized medical dataset. Wang et al. [39] used a weak-supervised method for the classification and detection of eight chest diseases presented on ChestX-ray8 dataset. The used method showed higher results for the detection of large abnormalities compared to small ones, achieving an average AUC of 80.30%. Using ChestX-ray8 dataset and based on the results attained in [39], Yao et al. [148] applied an LSTM (Long Short-Term Memory) based method to show the dependency inter-labels after extracting the features of the diseases using a DenseNet model. This approach obtained High performance, achieving an average AUC of 79.80% Rajpurkar et al. [102] employed a DenseNet-121 model (CheXNet) using the ChestX-ray14 dataset. The model achieved state-of-the-art results using binary relevance classification for the 14 diseases of the used dataset, achieving an average AUC of 84.11%. Ait Nasser and Akhloufi [64] performed an ensemble learning of three different DCNN models (Xception, DenseNet-201, and EfficientNet-B5) to classify CXR images into three classes (normal, lung disease, and heart disease). A dataset of 26,316 CXR images was created by merging images from VinDr-CXR and CheXpert datasets. Data-augmentation techniques were applied to increase the number of samples and to prevent the overfitting. The proposed ensemble learning approach showed high performance when used with data-augmentation techniques, achieving an average AUC of 94.89%. Kumar et al. [149] used the ChestX-ray14 dataset to classify the 14 diseases using a cascade neural network. DenseNet-161 model was used for a binary relevance classification. To prevent the bias due to imbalanced data, under-sampling and over-sampling techniques were applied. The introduced model reached competitive performance to state-of-the-art. It achieved an average AUC achieved of 79.50%. Zhao et al. [150] proposed a DCNN model with attention mechanism (AMDenseNet) to predict the presence of 14 chest diseases using CXR images from the Chest-Xray14 dataset. The model based on DenseNet-121 achieved a high average AUC of 85.37% outperforming the state-of-the-art works, such as [39,149]. Kim et al. [151] used EfficientNet-V2M with transfer learning as end-to-end approach to classify CXR images into three classes (normal, pneumonia, and pneumothorax). Preprocessing techniques were applied on images form ChestX-ray14 dataset before generated feeding data to the used model. EfficientNet-V2M achieved impressive results with a mean ACC of 82.15%, an average SEN of 81.40% and a mean SPE of 91.65%. The model achieved an average ACC of 82.20% when experimented on a dataset with four classes (normal, pneumonia, pneumothorax, and tuberculosis) collected from Cheonan Soonchunhyang University Hospital (SCH) [152]. Blais and Akhloufi [153] employed multiple models using binary relevance for the detection of chest diseases from CheXpert dataset. The Xception DCNN model performed better than other models when used with Adam optimizer, achieving a mean AUC of 95.87% on 6 diseases and 94.90% on the 14 diseases of the used dataset. Table 11 gives an overview of works developed for multi-disease CXR classification.

## 5. Discussion

This section is split into two main parts. The first part is related to data labeling and preprocessing, in which we discuss a number of limitations encountered by most researchers, and propose alternative techniques to address these challenges. The second part deals with the importance of interpretability of models in medical analysis, which is less considered in most of published papers.

### 5.1. Data Preprocessing

The publication of labeled datasets is fundamental to advancing the state of the art in medical analysis and driving research into new methods and techniques. However, collecting well-labeled datasets is costly and time-consuming. Most existing datasets are labeled using automatic labelers based on keywords matching methods (e.g., CheXpert and ChestX-ray14 datatsets) or NLP methods (e.g., CheXbert [154]) to extract labels from free-text radiology reports. The use of automatic labeling techniques can generate large-scale labels in less time, but they can produce errors in the labels [48] for a variety of reasons, such as lack of details in medical reports (some abnormalities may not be mentioned) and the performance of the algorithm used for labeling may be poor, which may lead to failures [112]. To cope with this situation, Calli et al. [155] trained DL models for the classification of emphysema using ChestX-ray14 dataset with noisy labels. They proved that the existence of a reasonable amount of uncertainty and erroneous labels in the training set has no impact on the performance of DCNN models. Another experiment was performed by Rolnick et al. [156] by training DCNN models on corrupted data from MNIST, CIFAR, and ImageNet. High performance was obtained despite the fact that the data were corrupted. Using a gold-set of test data labels is highly recommended for testing and evaluating the qualities of models. Another point to consider is label-dependencies, which has an important impact on medical diagnosis, as it may lead to a loss of vital information if not taken into account. This aspect (label-dependencies) has been overlooked in most studies, particularly when it comes to multi-disease classification.

It can be seen from Table 1 that datasets we presented in this literature have thousands of CXR images with multiple abnormalities collected from several hospitals. The majority of them are imbalanced, especially when it comes to normal cases (healthy), which in some datasets constitute more than half of the total number of images. On the other hand, the number of CXR images for some abnormalities is extremely limited. For example, VinDr-CXR dataset has 10,606 images for normal cases and 58 images for pneumothorax. Training a model on an imbalanced dataset may lead to overfitting, which impacts the performance of the model. Therefore, preprocessing techniques were used by most of the studies, and they proved to be effective, especially when used with deep learning techniques that require the existence of a large amount of data. For data-augmentation, the recent papers employed traditional techniques [64], where others used GANs to generate artificial CXR images. This technique (GAN) is becoming widely used by researchers because of the results obtained, particularly in the medical field [157,158,159].

The availability of large strongly-labeled CXR datasets will also help in developing CAD systems capable of integrating the degree of infection by a specific disease. This quantitative dimension in disease detection is not very present in research. In addition, the majority of presented works used only frontal CXR, whereas lateral CXR can provide more detail on disease features. It was showed that lateral CXR can show up to 15% of lung cancer features hidden by other structures on frontal CXR [160].

Access to data that belong to external hospitals remains limited due to medical privacy rules. Therefore, in the great majority of research, the same datasets are used for training and testing models. However, the most effective models tend to perform weaker when tested on external data, as shown by Hwang et al. [161] who investigated the performance of a DCNN model for the classification of CXR images from an emergency department. This proved that the efficiency of the model decreased with at least 2% in terms of AUC. This may be related to several factors, including the quality, the resolution, the size and the content of images (deformed lungs, collapsed parts of the lungs, etc.). Limited access to data from external institutions is a real challenge for the research community. For this, the best technique suggested is what is called federated learning. This technique allows using data from several hospitals with a high level of security and without violating privacy rules. For COVID-19 detection, the major concern is the availability of large public datasets. It can be seen from Table 2 that there is a lack of labeled images for COVID-19, which led some researchers to merge images from available sources to increase the amount of data samples. Where others used federated learning to train models on data from multiple institutions without breaching the data privacy. Feki et al. [162] used federated learning for COVID-19 detection. Their results were competitive to what is obtained when models were trained on centralized data. Liu et al. [163] compared the performance of models used with and without federated learning for COVID-19 detection. The obtained results may inspire the research community to spend more time on federated learning.

### 5.2. Models Interpretability

ML algorithms were among the first automatic methods used to detect diseases on medical images. Multiple papers showed the potential of ML models on small datasets [23,24]. The process of detecting anomalies using ML is technically decomposed into multiple parts to be resolved first, before combining the results in the final step. For example, the extraction of features process is usually performed manually in a separated part using specific algorithms before applying a classifier to provide predictions. This requires the intervention of specialists to select features from the input data before feeding the models. This represented a challenge as the performance of ML models decreases when used on large datasets. On the other hand, DL showed impressive results, especially with the availability of large datasets and high computation resources, which helped to overcome the limitations of ML.

Disease detection using DL techniques is an end-to-end process, where the models take an image as input and provide a prediction as output. Feature extraction is performed incrementally through the hidden layers. Despite the successes of DL techniques, they are considered to be black boxes whose performance or failure is hardly explainable. This process of explainability and interpretability of the models was not taken into account by the majority of studies. However, explainability is not a purely technical concern, but involves a multitude of medical, legal, moral, and social concerns that require careful consideration. It is very important to highlight the features that the model extracts to provide a decision, as performed by Singh et al. [164] and Chetoui et al. [140] who proposed explainable approaches for COVID-19 detection based on Grad-CAM algorithm [141] to output the heatmaps and show how the most interesting findings were achieved by the models.

Providing explainable results gives radiologists more confidence to implement CAD systems in medical analysis operations. Multiple commercial products for the CXR image analysis are available, such as Samsung Healthcare system (Auto Lung Nodule Detection) [165] which performs the detection and localization of lung nodule, Siemens Healthineers system [166] (AI-Rad Companion Chest X-ray) which performs the localization of multiple diseases (pleural effusion, pneumothorax, lesion, and other), and Oxipit system [167] (ChestEye CAD) which is a CXR support tool for radiologists. All those commercial systems detect and localize chest diseases by providing reports and heatmaps visualization, and they are all approved by Food and Drugs Administration (FDA).

Increasingly, researchers are exploring DL techniques on all medical imaging modalities, especially chest radiography (CXR), which is the most cost-effective modality with large publicly available datasets collected over the years by different institutions. The release of CXR datasets was a good opportunity for researchers to discover the multiple uses of DL in order to develop efficient CAD systems for chest disease detection. These advances are not intended to replace the radiologist, but to work hand-in-hand with them to improve the performance and speed up the diagnosis.

Based on works presented in this review, we noticed that some DL models were more used than others for the detection of different diseases. For instance, several architectures based on fine-tuned ResNet models were proposed for the classification of COVID-19, pneumonia, tuberculosis, and pulmonary nodule. ResNet models showed promising performances by achieving high scores on different datasets. We also found that transfer learning techniques were widely present in multiple experiments. They allowed obtaining higher results especially in the case of scarcity of data [129,132,134].

## 6. Conclusions

Deep learning (DL) has become one of the most effective technologies for analyzing and processing medical images. It offers a plethora of methods and solutions to develop tools that help clinicians predict the risk of diseases and prevent them at an early stage. Medical image processing using DL techniques is a very promising research area where the fields of medicine and computer science intersect. In this review, we summarize research in the following direction. First, the most widely used and publicly available X-ray datasets were described in detail. Second, the performance metrics for classification and segmentation were briefly introduced and various preprocessing techniques, such as data-augmentation, image enhancement, lung segmentation, and bone suppression were presented and discussed by showing their impact on the performance of DL models. Third, recently published DL architectures were introduced with more focus on chest disease classification rather than localization, and this is a point that can be further developed. Finally, the main challenges published works have faced were covered and an in-depth discussion of alternative solutions to be considered in future works was held.

In a future work, we would like to highlight the power of up-to-date proposed transformer models for chest disease detection. Many transformers’ architectures have been published recently, showed promising results in different fields. We foresee to elaborate an in-depth comparison between these architectures and popular DCNN models to show the achievements of these two approaches, their impact on the medical field and the main challenges to be addressed. A study of new advances on the subject of explainability and interpretability of models can also be conducted.

## Figures and Tables

**Table 4:** Overview of different enhancement techniques for CXR images.

Ref.	Dataset	Technique
[75]	COVID-19-Radiography Database	Gabor filtering
[76]	Shenzhen	CLAHE, unsharp masking, and high frequency emphasis filtering
[77]	RSNA-Pneumonia-CXR and BIMCV-COVID19+	HE, CLAHE, image invert, gamma correction, and BCET
[78]	RSNA-Pneumonia-CXR	Unsharp mask, CLAHE, and HE
[80]	ChestX-ray14	HE and CLAHE
[81]	Custom dataset	CLAHE with normalization function
[82]	CXR images from Montgomery, ChestX-ray14, and Shenzhen	Contrast adjustment

**Table 5:** Overview of different segmentation techniques for CXR images.

Ref.	Dataset	Technique
[83]	COVID Chest X-ray	U-Net
[84]	379 CXR images from JSRT and Montgomery	FCN
[85]	ChestX-ray14	U-Net
[86]	JSRT	Pix2pix
[87]	CXR images from Shenzhen, Montgomery, and JSRT	ARSeg with attention mechanism
[88]	JSRT	SCAN
[89]	Montgomery and JSRT	U-Net

**Table 7:** Summary of different DL architectures used for pneumonia detection.

Ref.	Dataset	Model	Results
[9]	Pediatric-CXR	CNN model with and without data-augmentation	ACC = 83.38%
[63]	Pediatric-CXR	Custom DCNN model with transfer learning	ACC = 92.80% SEN = 93.20% SPE = 90.10% AUC = 96.80%
[98]	Pediatric-CXR	18-layer deep sequential CNN model	ACC = 94.39% SEN = 99.00% SPE = 86.00%
[99]	Pediatric-CXR ChestX-ray8	Swin transformer with a fully connected layer	ACC = 97.20% ACC = 87.30%
[100]	Pediatric-CXR	ResNet50 with attention mechanism	ACC = 95.73%
[101]	RSNA-Pneumonia-CXR	Inception-V4 with transfer learning	ACC = 94.00%
[102]	ChestX-ray14	CheXNet model (121-layer CNN)	AUC = 76.80%
[103]	Pediatric-CXR RSNA-Pneumonia-CXR	Ensemble learning of three DCNN models (GoogleNet, ResNet-18, and DenseNet-121)	ACC = 98.81% ACC = 86.86%
[104]	X-viral dataset (5977 viral-pneumonia and 37,393 non-viral pneumonia images) and X-Covid dataset (106 COVID-19, 107 normal)	Confidence-aware anomaly detection (CAAD) model	AUC = 83.61% SEN = 71.70%
[105]	Pediatric-CXR	DCNN with and without dropout and data-augmentation	ACC = 90.00%
[106]	Pediatric-CXR	Custom DCNN model from scratch	ACC = 93.73%

**Table 8:** Summary of different DL techniques for pulmonary nodule detection.

Ref.	Dataset	Model	Results
[6]	17,211 CXRs for training (augmented to 600,000 training images) and 10,285 CXRs for testing (1483 CXRs with lung cancer)	ResNet-50 and ResNet-101	AUC = 73.20% SEN = 76.80%
[37]	411 CXRs, 257 with annotated pulmonary nodules and 154 normal	RetinaNet with ResNet-101 as backbone	AUC = 87.00%
[111]	JSRT dataset	ResNet-50	SEN = 92.00% SPE = 86.00%
[109]	13,710 normal and 3500 lung nodules CXR images for training, 800 CXR images for testing. Images were obtained in four hospitals between 2015 and 2017 by two expert radiologists	ResNet-50	ACC = 70.30%
[110]	180 segmented CXR images from JSRT (90 nodule and 90 non-nodule images)	Custom DCNN model with data-augmentation techniques	AUC = 86.67%
[112]	745,479 CXR scans acquired from the historical archives of Guy’s and St. Thomas’ NHS Foundation Trust in London from January 2005 to March 2016	Convolutional network with attention feedback model based on VGG-13 architecture	ACC = 85.00% SEN = 78.00% PRE = 92.00% F1-score = 85.00%
[113]	1881 CXRs (958 normal, 923 pneumoconiosis) obtained from the PACS at pekin University Third Hospital	Fine-tuned Inception-V3	AUC = 87.80%
[114]	JSRT dataset	Custom DCNN with lung field segmentation, bone suppression, and full features fusion technique	ACC = 99.00%
[115]	2440 images (2088 with nodule and 352 normal) collected from CheXpert [44]	Mask R-CNN and RetinaNet	SEN = 95.60%

**Table 9:** Summary of different DL architectures used for tuberculosis detection.

Ref.	Dataset	Model	Results
[11]	Shenzhen and Indiana	InceptionV3 with transfer learning	AUC = 98.45% SEN = 72.00% SPE = 82.00%
[60]	Montgomery, Shenzhen, and KIT	Custom DCNN model based on AlexNet	AUC = 96.40% ACC = 90.30%
[116]	Images from three datasets (Montgomery, a dataset created by different institutes under the ministry of health of the Republic of Belarus, and a kaggle repository).	Custom DCNN model called TBXNet	ACC = 99.17%
[117]	Shenzhen	VGG-16 with coordinate attention mechanism (VGG16-coordattention)	AUC = 97.71% ACC = 92.73% PRE = 97.71%
[118]	Montgomery and Shenzhen	ConvNet model trained from scratch	AUC = 87.00% SEN = 87.00% PRE = 88.00%
[119]	Shenzhen	AlexNet and GoogleNet	AUC = 99.00% SEN = 97.30% SPE = 100%
[120]	Custom dataset of 3500 TB and 3500 normal CXR images acquired from different open-access datasets such as Montgomery and Shenzhen datasets	DenseNet-201 model using transfer learning	ACC = 98.60% PRE = 98.57% SEN = 98.56% SPE = 98.54% F1-score = 98.56%
[121]	A dataset of 7000 CXR images (3500 normal and 3500 TB) [120]	Ensemble learning of three DCNN models (ResNet-50, VGG-19, and DenseNet-121)	ACC = 99.75%
[122]	Montgomery and Shenzhen	DCNN model with seven convolutional layers and three fully connected layers	ACC = 82.09%
[123]	Shenzhen and Montgomery	DenseNet-121	AUC = 99.00% AUC = 84.00%
[124]	Montgomery and Shenzhen	Ensemble learning of DCNN models (GoogleNet, ResNet, and VGGNet)	ACC = 84.60% AUC = 92.60%
[125]	Montgomery and Shenzhen	VGG-16	ACC = 86.74% AUC = 92.00%
[126]	JSRT	DCNN model (ResNet) with a class decomposition approach	ACC = 99.80% SEN = 98.00% SPE = 99.00%

**Table 11:** Overview of different DL architectures for multiple disease detection.

Ref.	Dataset	Diseases	Results
[39]	ChestX-ray8	8 thoracic diseases	AUC (Mean) = 80.30%
[102]	ChestX-ray14	14 thoracic diseases	AUC (Mean) = 84.20%
[140]	Merged 9 datasets	Normal Pneumonia COVID-19	AUC (Mean) = 97.00%
[145]	CXR images from CheXpert	Cardiomegaly (CA) Pulmonary nodule (PUN)	AUC (CA) = 92.00% AUC (PUN) = 73.00%
[146]	93 CXR images collected from Sheba Medical Center	Pleural Effusion (PE)Cardiomegaly (CA)Normal (N)Abnormal (AB)	AUC (PE) = 93.00% AUC (CA) = 89.00% AUC (N Vs AB) = 79.00%
[147]	35,038 CXR images exported from the PACS repository	Normal (N)Cardiomegaly (CA)Pleural effusion (PE)Pulmonary edema (E) Pneumothorax (PN) Consolidation (CO)	AUC (N) = 96.40% AUC (CA) = 87.50% AUC (PE) = 96.20% AUC (E) = 86.80% AUC (PN) = 86.10% AUC (CO) = 85.00%
[64]	A consolidated dataset of 26,316 CXR images collected from CheXpert and VinDr-CXR	Lung diseaseHeart diseaseNormal (N)	AUC (Mean) = 94.89%
[149]	ChestX-ray14	14 thoracic diseases	AUC (Mean) = 79.50%
[150]	ChestX-ray14	14 thoracic diseases	AUC (Mean) = 85.37%
[151]	ChestX-ray14	Normal Pneumonia Pneumothorax	ACC (Mean) = 82.15%
[153]	CheXpert	14 thoracic diseases	AUC (Mean) = 94.90%

## Data Availability

Not applicable.

## Abbreviations

The following abbreviations are used in this manuscript:

CXR	Chest X-ray Radiography
CT	Computed Tomography
MRI	Magnetic Resonance Imaging
CAD	Computer Aided Detection
ML	Machine Learning
DL	Deep Learning
NN	Neural Network
DCNN	Deep Convolutional Neural Network
NLP	Natural Language Processing
FCN	Fully Connected Network
FCL	Fully Connected Layer
GAN	generative adversarial network
Grad-CAM	Gradient-Weighted Class Activation Mapping
ROI	Region of Interest
WHO	World Health Organization
FDA	Food and Drugs Administration
COPD	Chronic Obstructive Pulmonary Disease
TB	Tuberculosis
SVM	Support Vector Machine
KNN	K-nearest Neighbors
ACC	Accuracy
PRE	Precision
SEN	Sensitivity
SPE	Specificity
AUC	Area Under Curve
